# Developmental Outcomes Among Young Children With Congenital Zika Syndrome in Brazil

**DOI:** 10.1001/jamanetworkopen.2020.4096

**Published:** 2020-05-05

**Authors:** Anne C. Wheeler, Danielle Toth, Ty Ridenour, Lucélia Lima Nóbrega, Raíne Borba Firmino, Claudia Marques da Silva, Pollyanna Carvalho, Douglas Marques, Katherine Okoniewski, Liana O. Ventura, Donald B. Bailey, Camila V. Ventura

**Affiliations:** 1 Center for Newborn Screening, Ethics, and Disability Studies, Research Triangle Institute International, Research Triangle Park, North Carolina; 2 Rehabilitation Center Menina dos Olhos, Altino Ventura Foundation, Recife, Brazil

## Abstract

**Importance:**

Although birth defects in children with congenital Zika syndrome (CZS) are expected to result in significant intellectual disabilities, the extent of delay and profiles of development have yet to be fully described.

**Objectives:**

To describe the neurodevelopmental profiles of children with CZS and to test whether prenatal and postpartum characteristics were associated with the severity of developmental delays.

**Design, Setting, and Participants:**

This is a case series of the trajectories of developmental, behavioral, and medical needs of 121 young children with CZS who were assessed at a specialized rehabilitation center in Recife, Brazil, beginning in January 2018 as part of 5-year longitudinal study. Children were included if they had serologic confirmation of Zika virus and met clinical criteria accompanied by parental report of suspected exposure to Zika virus during pregnancy.

**Exposures:**

Prenatal Zika virus exposure.

**Main Outcomes and Measures:**

The Brazilian version of the Bayley Scales of Infant and Toddler Development, Third Edition, was administered by trained assessors as part of an initial comprehensive assessment battery. Caregiver interviews and medical record reviews were conducted to gather basic demographic information and medical comorbidities. Linear regression was used to identify potential factors for development.

**Results:**

The sample included 121 young children (mean [SD] age, 31.2 [1.9] months; 61 [50.4%] girls). At age approximately 2.5 years, nearly all children in this sample demonstrated profound developmental delays across all domains of functioning, with a mean (SD) developmental age equivalent to approximately 2 to 4 months (eg, cognitive domain, 2.24 [3.09] months; fine motor subscale, 2.15 [2.93] months; expressive language subscale, 2.30 [2.52] months). A relative strength was found in receptive language, with scores on this scale significantly higher than most other domains (eg, cognition: *t* = 3.73; *P* < .001; fine motor: *t* = 6.99; *P* < .001). Head circumference at birth was the single strongest factor associated with outcomes across all developmental domains (eg, cognitive: β = 1.41; SE, 0.67; *P* = .04; fine motor: β = 1.36; SE, 0.49; *P* = .007).

**Conclusions and Relevance:**

The findings of this study provide important information regarding the severity of disability that these children and their families will experience. The findings also establish an initial point from which to monitor developmental trajectories, medical comorbidities (eg, seizures), effectiveness of interventions, and cumulative consequences on families.

## Key Points

**Question:**

What are the observed neurodevelopmental sequelae among young children with congenital Zika syndrome (CZS) identified during the Zika virus outbreak in Brazil from 2015 to 2016?

**Findings:**

In this case series of 121 young children, nearly all children with CZS demonstrated profound developmental delays at age 2 to 3 years across all domains of functioning, with a relative strength in receptive communication. Severity of microcephaly at birth was the only significant factor associated with the severity of delays.

**Meaning:**

In this study, most children diagnosed with CZS had profound developmental delays; however, there was variability within their developmental profile, providing direction for intervention.

## Teaser

This case series describes the neurodevelopmental profiles of children with CZS and tests whether prenatal and postpartum characteristics were associated with the severity of developmental delays.

## Introduction

In 2015, Brazil experienced a widespread epidemic of Zika virus (ZIKV) infection, which ultimately spread across the Americas, with 87 countries and territories reporting autochthonous transmission.^1^ Although estimates suggest a seroprevalence of more than 60% in some regions,^2^ it is difficult to estimate exposure given that 60% to 80% of infections are asymptomatic^3^ and adult symptoms were typically mild and nonspecific. However, fetal exposure has had severe consequences for a subset of infants. Early reports focused on findings of microcephaly.^4^ However, it is now clear that congenital Zika syndrome (CZS) has variable presentation, severity, and prognosis.^5^ Congenital Zika syndrome differs from other congenital infections in the 5 following ways: “severe microcephaly with partially collapsed skull; thin cerebral cortices with subcortical calcifications; macular scarring and focal pigmentary retinal mottling; congenital contractures; and marked early hypertonia and symptoms of extrapyramidal involvement.”^5,6^ According to the Pan American Health Organization, 31 countries reported microcephaly and/or central nervous system complications associated with ZIKV infection.^5^ Approximately 3000 children were born with CZS during the 2015 outbreak in Brazil, mostly in the northeast.^7^

At least 2 questions must be answered regarding the consequences of prenatal ZIKV exposure. First, what are the effects on any exposed fetus? Second, what is the prognosis for those with obvious features of CZS at birth? Our study addresses the second question by assessing a sample of children who had prenatal ZIKV exposure and exhibited anomalies at birth not otherwise explained. These children are at highest risk for severe, lifelong consequences on development and function. Our broad goals were to characterize consequences and symptoms over time, identify potentially useful interventions for children and families, and document burden for families and costs to society.

Studies to date have provided important information about ZIKV exposure, including ocular manifestations,^8^ neurological findings,^9^ epileptogenic activity, arthrogryposis, and hearing loss.^10^ Several studies have detailed functional impairments and neurodevelopmental skills in small samples, suggesting a high likelihood of severe to profound disabilities. Two early studies^10,11^ reported profound delays as measured by a parent-report tool. A cross-sectional study of eight children (mean age, 21.1 months) with CZS using the Bayley Scales of Infant and Toddler Development, Third Edition (BSID-III),^12^ reported that all children scored at or close to the floor on cognitive and motor domains. Another study^13^ described developmental outcomes for 35 children with CZS, reporting below average BSID-III scores and abnormal brain findings confirmed by brain imaging. A larger study of 146 children aged 7 to 32 months whose mothers had confirmed gestational ZIKV infection^14^ found that 40% had mild delays (<1 SD below BSID-III mean) and 12.3% had moderate to severe delays in at least 1 functional domain of the BSID-III. However, most children in this study who had exposure did not exhibit obvious anomalies at birth. To our knowledge, no published studies have explored profiles of development in large cohorts of children with obvious clinical features of CZS at birth.

In 2016, we launched a longitudinal study of children with CZS and their families. This study has 4 unique features. First, we have a well-characterized sample of children with laboratory-confirmed congenital ZIKV infection and clinical manifestations. Second, we are conducting a comprehensive battery of assessments over a 5-year period, documenting overall function, identifying secondary complications, and characterizing emerging profiles of strengths and weaknesses. Third, we are assessing the evolving consequences on families.^15^ Finally, by selecting a sample of children who are receiving a range of services we will be able to identify and measure existing strategies that support child development and family adaptation.

This article reports baseline developmental assessments of children at age approximately 30 months to determine overall developmental status, identify areas of relative strengths and delays, and determine whether selected factors (ie, sex, age, head circumference at birth, gestational age, birth weight, and household income) were associated with overall function or developmental profiles.

## Methods

Participants were 121 toddlers with CZS receiving clinical services at a rehabilitation center in Recife, Brazil. Primary caregivers provided written informed consent while present at the rehabilitation center after being read aloud the consent forms. This study was approved by the institutional review board at the Altino Ventura Foundation (FAV) in Pernambuco, Brazil. Study data were collected and managed using REDCap electronic data capture tools.^16^ Reporting conforms to the Strengthening the Reporting of Observational Studies in Epidemiology (STROBE) reporting guideline.

The sample was limited to children with serologic confirmation of ZIKV, obtained by the detection of immunoglobulin M antibody capture enzyme-linked immunosorbent assay or real-time reverse transcription–polymerase chain reaction testing from cerebrospinal fluid (collected at age 3 months or younger), who met clinical criteria accompanied by parental report of suspected exposure to ZIKV during pregnancy.^17^ Symptoms reported by mothers included rash, fever, joint pain, conjunctivitis, headaches, and ocular pain during pregnancy. Before the 2016 outbreak of ZIKV, assays to detect ZIKV were only available for research or from the US Centers for Disease Control and Prevention.^18,19,20^ Inclusion in this study was limited to children who presented with clinical abnormalities associated with CZS (ie, microcephaly, central nervous system damage, subcortical calcifications, ocular damage, congenital contractures, hypertonia extrapyramidal involvement)^4^; had serology confirmation ruling out toxoplasmosis, rubella, cytomegalovirus, herpes, syphilis, or HIV^4^; and had neuroimaging findings consistent with CZS brain abnormalities. Microcephaly and severe microcephaly were defined based on International Fetal and Newborn Growth Consortium for the 21st Century fetal growth standards.^21^

Children were seen at the clinic for a developmental assessment using the Brazilian Portuguese version of the BSID-III,^22^ a widely used norm-referenced measure of development that spans 3 domains (ie, cognitive, language, and motor), with 5 subdomains (ie, cognitive, receptive language, expressive language, fine motor, and gross motor). Three scores are provided for each domain, as follows: raw scores, age-equivalent scores, and scaled scores (mean [SD], 10 [3]; range, 1-19). Standard scores, with a mean of 100 and an SD of 15, can be computed for the cognitive scale, and composite scores for language (combining the receptive and expressive scales) and motor (combining the fine and gross motor scales) scales.

We used age-equivalent scores to explore profiles of strengths and weaknesses across the 5 subdomains. Age-equivalent scores allow comparison of performance on domains (in contrast to raw scores, for which ranges vary). However, because of the lack of variability in norm-referenced scores, we used domain raw scores for correlation and regression analyses. Raw scores reflect the number of items a child can complete in contrast to scaled scores, which provide a comparison to the normed population. While raw scores are generally not used in analyses because of varying expectations based on age, the children in our sample were within a relatively small age span (mean [SD] age, 31.2 [1.9] months) and thus raw scores provided a more robust method for exploring variability within the sample.

Children were assessed by trained research assistants with experience working with children with CZS and similar disabilities. They received extensive training on BSID-III administration and acceptable accommodations for children with severe disabilities. All administrations were videotaped and coded for fidelity by an independent psychologist (K.O.) with expertise in the BSID-III. Errors in scoring or administration were corrected with feedback to the research assistants for future administrations. No errors were reported that compromised the validity of the data. The BSID-III was administered using standard procedures, with accommodations for the child’s visual or motor impairments, as suggested by the manual.^22^ For example, for children with light vision only, overhead lights were turned off and a flashlight was used to provide contrast. Parents or guardians were present during the BSID-III administration. Demographic information and medical histories were obtained through parent or caregiver interview and review of medical records.

### Statistical Analysis

Descriptive analyses included frequencies, means, SDs, and ranges, calculated for general characteristics including age, sex, gestational age, type of delivery, and weight and head circumference at birth. Descriptive analyses of the total raw scores and age-equivalent scores were also included across the 5 developmental domains of the BSID-III.

Comparison of means tests (*t* tests) were conducted to assess differences in sex among the 5 domains. Correlation analyses were performed to examine the association of independent variables with raw scores. Pearson correlation coefficients between developmental scores and demographic variables (ie, age, maternal age, household income) and clinical variables (ie, birth weight, gestational age, head circumference at birth) were included in the analysis. A stepwise multivariate linear regression was calculated to identify potential factors associated with development, including sex, age, head circumference at birth, gestational age, birth weight, and household income. All analyses were performed using the SAS version 9 (SAS Institute). Statistical significance was set at *P* < .05, and all tests were 2-tailed.

## Results

The 121 children had a mean age at assessment of 31.2 (1.9) months, with 58 (49.6%) boys and 61 (50.4%) girls. A total of 99 children (81.8%) had microcephaly at birth, 74 (61.2%) of whom were classified as severe (>3 SDs below mean head circumference) (Table 1). Means, SDs, and ranges for standard scores, raw scores, and age-equivalent scores are provided in Table 2. Nearly all (106 [87.6%] to 118 [97.5%]) participants scored at the floor of at least 1 scale. However, there was variability in raw scores and age-equivalent scores. For example, a standard score of 55 for the cognitive domain was assigned to children with raw scores ranging from 1 to 42. Table 3 and Table 4 provide results for correlation and regression analyses testing factors’ associations with outcomes. Comparison tests revealed no differences by sex of child on any BSID-III scale or control variable. Microcephaly at birth (cognitive: *r* = −0.271; *P* = .004; receptive communication: *r* = −0.226; *P* = .02; fine motor skills: *r* = −0.201; *P* = .04; gross motor skills: *r* = −0.205; *P* < .001), severity of microcephaly (cognitive: *r* = −0.332; *P* < .001; fine motor skills: *r* = −0.260; *P* < .001; gross motor skills: *r* = −0.230; *P* = .02), head circumference at birth (cognitive: *r* = 0.376; *P* < .001; receptive communication: *r* = 0.224; *P* = .02; expressive communication: *r* = 0.233; *P* = .01; gross motor skills: *r* = 0.236; *P* = .01), and birth weight (cognitive: *r* = 0.295; *P* = .002; receptive communication skills: *r* = 0.246; *P* = .009; expressive communication skills: *r* = 0.230; *P* = .02; fine motor skills, *r* = 0.258; *P* = .006) were all significantly correlated with at least 3 developmental domains. However, within the regression models, only head circumference at birth was significant across cognitive (β = 1.41; SE, 0.67; *P* = .04) and fine motor (β = 1.36; SE, 0.49; *P* = .007) scales, and maternal age and birth weight were associated with receptive language scores (maternal age at birth: β = 0.11; SE, 0.05; *P* = .02; birth weight: β = 2.29; SE, 0.92; *P* = .02). More detailed results are described based on each developmental domain below.

**Table 1.** Baseline Characteristics of the Study Population

Characteristic	Children, No. (%)
Sex, No.	121
Girls	63 (52.1)
Boys	58 (47.9)
Age, No.	177
Mean (SD), cm	31.5 (2.85)
Head circumference at birth, No.	160
Mean (SD), cm	28.76 (1.88)
Microcephaly at birth, No.	110
No	11 (10.0)
Yes	99 (90.0)
Severity of microcephaly at birth, No.	110
None	11 (10.0)
>2 to <3 SDs below mean	25 (22.7)
>3 SDs below mean	74 (67.3)
Type of delivery, No.	106
Vaginal	54 (50.9)
Cesarean	52 (49.1)
Birth weight, No.	159
Mean (SD), kg	2.70 (0.54)
Gestational age at birth	160
Mean (SD), wk	38.13 (2.05)
Preterm, No.	103
Yes	18 (17.5)
No	85 (82.5)
Maternal age at birth, No.	175
Mean (SD), y	26.85 (7.22)
Trimester presenting Zika symptoms, No.	91
First	44 (48.4)
Second	34 (37.4)
Third	9 (9.9)
No symptoms reported during pregnancy	4 (4.4)
Seizure severity, No.	120
Mild	87 (81.3)
Moderate	19 (17.8)
Severe	1 (0.9)
Visual impairment, No.	75
Normal	23 (30.7)
Low	16 (21.3)
Moderate	27 (36.0)
Severe	9 (12.0)
Monthly household income, No.	120
<Minimum wage	6 (5.0)
Minimum wage	62 (51.7)
1 to <2 times minimum wage	42 (35.0)
2 to <3 times minimum wage	7 (5.8)
>3 times the minimum wage	3 (2.5)
Maternal education, No.	86
Incomplete primary education	21 (25.0)
Primary education	5 (5.9)
Incomplete secondary education	9 (10.7)
Secondary education	38 (45.2)
Vocational school	2 (2.4)
Incomplete university	5 (5.9)
University	3 (3.6)
Postgraduate education	2 (6.7)
Illiterate	0
Functionally illiterate	1 (1.2)
Marital status, No.	121
Single	24 (20.0)
Married	37 (30.8)
Civil union or long-term partnership	4 (3.3)
Separated	2 (1.7)
Divorced	2 (1.7)
Widowed	1 (0.8)
Other or not reported	51 (42.1)
Neurological findings, No.	177
Decreased brain volume	71 (59.2)
Enlarged lateral ventricles	91 (75.8)
Enlarged fourth ventricle	17 (14.4)
Hydrocephaly	4 (3.4)
Reduced sulcation	9 (7.5)
Lysencephaly	26 (21.7)
Agyria	12 (10)
Pachygyria	29 (24.2)
Parenchymal calcifications	112 (94.1)
Cortical calcifications	58 (49.2)
Subcortical calcifications	73 (61.3)

**Table 2.** Domain Scores on the Bayley Scales of Infant and Toddler Development, Third Edition

Score	Cognitive	Composite		Language		Motor	
		Language	Motor	Receptive	Expressive	Fine	Gross
Standard scores							
Mean (SD) [sample range]	55.23 (1.75) [55-70]	47.71 (2.89) [47-71]	46.26 (1.83) [46-64]	NA^a^	NA^a^	NA^a^	NA^a^
Floor score (No. [%] at floor)	55 (118 [97.5])	47 (106 [87.6])	46 (116 [95.9])	1^b^	1^b^	1^b^	1^b^
Raw scores							
Mean (SD) [sample range]	10.47 (9.75) [0-54]	NA^a^	NA^a^	6.69 (3.19) [0-24]	4.60 (3.39) [0-21]	6.36 (6.72) [0-35]	9.89 (8.58) [0-47]
Raw scores resulting in floor score	1-42	NA^a^	NA^a^	NA^a^	NA^a^	NA^a^	NA^a^
Scaled scores							
Mean (SD) [sample range]	1.05 (0.35) [1-4]	NA^a^	NA^a^	1.17 (0.64) [1-7]	1.08 (0.47) [1-5]	1.05 (0.39) [1-5]	1.03 (0.20) [1-3]
Age-equivalent score							
Mean (SD) [sample range]	2.24 (3.09) [0.16-19]	NA^a^	NA^a^	3.02 (2.89) [0.16-21]	2.59 (2.96) [0.16-20]	2.15 (2.93) [0.16-20]	2.29 (2.52) [0.16-15]

Abbreviation: NA, not applicable.

^a^ Scores do not exist for this scale.

^b^ The floor score for the individual scales is 1.

**Table 3.** Correlations Between Demographic Variables and Clinical Characteristics and Raw Scores on Bayley Scales of Infant and Toddler Development, Third Edition, Among Children With Congenital Zika Syndrome

Variable^a^	*r*				
	Cognitive Scale	Communication scale		Motor scale	
		Receptive subscale	Expressive subscale	Fine subscale	Gross subscale
Age	−0.08343	0.02188	−0.00733	0.02619	−0.08057
Sex	−0.08831	−0.07654	−0.06824	−0.00143	−0.04138
Mother’s age at birth	0.14190	0.20920	0.05100	0.12694	0.01981
Microcephaly diagnosis at birth	−0.27134^b^	−0.22553^b^	−0.12560	−0.20084^b^	−0.20522^b^
Severity of microcephaly	−0.33170^b^	−0.17247^c^	−0.18151^c^	−0.36008^b^	−0.22987^b^
Head circumference at birth	0.37632^b^	0.22432^b^	0.23261^b^	0.39464^b^	0.23586^b^
Birth weight	0.29509^b^	0.24564^b^	0.23002^b^	0.25770^b^	0.11855
Severity of seizures	0.04198	−0.07793	−0.02035	0.12475	0.00434
Frequency of seizures	−0.21787^b^	−0.06314	−0.09643	−0.08538	−0.04860
Preterm birth	−0.08243	−0.03935	−0.11851	−0.09588	−0.12651
Type of delivery	0.08848	−0.00969	0.02618	0.02610	0.10903
Gestational age at birth	0.02393	0.04566	0.09851	0.08025	0.01364
Trimester Zika symptoms informed	−0.06015	−0.00268	−0.07096	−0.01575	0.02642
Visual impairment	−0.11523	−0.14674	−0.10815	−0.11473	−0.12501
Amount of therapy and EI received monthly	−0.03411	−0.00990	−0.13248	−0.03419	−0.06066

Abbreviation: EI, early intervention.

^a^ Data were available for 99 to 177 infants.

^b^ *P* < .05.

^c^ *P* < .10.

**Table 4.** Logistic Regression Analysis of the Association of Demographic and Clinical Characteristics With Bayley Scales of Infant and Toddler Development, Third Edition, Scores

Characteristic	Model 1: demographic variables		Model 2: demographic variables and clinical variables		Model 3: demographic, clinical variables, and head circumference at birth	
	β (SE)	*P* value	β (SE)	*P* value	β (SE)	*P* value
**Cognitive scores**						
Sex						
Girls	−0.801 (1.92)	.68	−0.363 (1.845)	.84	−0.024 (1.831)	.99
Boys	0 [Reference]		0 [Reference]		0 [Reference]	
Age	−0.323 (0.537)	.55	−0.143 (0.516)	.78	−0.339 (0.519)	.52
Maternal age at birth	0.151 (0.136)	.27	0.133 (0.134)	.32	0.113 (0.135)	.40
Household income	0.942 (1.182)	.438	1.070 (1.132)	.35	0.486 (1.153)	.68
Birth weight	NA	NA	7.653 (2.122)	.001	4.055 (2.674)	.13
Gestational age	NA	NA	−0.916 (0.555)	.10	−0.869 (0.549)	.12
Head circumference at birth	NA	NA	NA	NA	1.405 (0.668)	.04
**Receptive communication scores**						
Sex						
Girls	−0.137 (0.648)	.83	−0.019 (0.629)	.98	0.071 (0.631)	.91
Boys	0 [Reference]	NA	0 [Reference]	NA	0 [Reference]	NA
Age	0.097 (0.182)	.59	0.147 (0.176)	.40	0.165 (0.179)	.36
Maternal age at birth	0.096 (0.046)	.04	0.095 (0.046)	.04	0.110 (0.047)	.02
Household income	0.084 (0.399)	.83	0.102 (0.386)	.79	0.154 (0.397)	.70
Birth weight	NA	NA	2.269 (0.723)	.002	2.292 (0.921)	.02
Gestational age	NA	NA	−0.222 (0.189)	.25	−0.198 (0.189)	.30
Head circumference at birth	NA	NA	NA	NA	−0.062 (0.230)	.79
**Expressive communication scores**						
Sex						
Girls	−0.235 (0.726)	.75	−0.103 (0.714)	.89	−0.012 (0.722)	.99
Male	0 [Reference]	NA	0 [Reference]	NA	0 [Reference]	NA
Age	−0.005 (0.204)	.98	0.023 (0.200)	.91	0.016 (0.205)	.94
Maternal age at birth	0.006 (0.051)	.91	−0.016 (0.052)	.76	−0.008 (0.053)	.88
Household income	0.359 (0.448)	.43	0.450 (0.438)	.31	0.430 (0.455)	.35
Birth weight	NA	NA	2.304 (0.822)	.006	1.989 (1.055)	.06
Gestational age	NA	NA	−0.131 (0.215)	.54	−0.111 (0.217)	.61
Head circumference at birth	NA	NA	NA	NA	0.088 (0.264)	.74
**Fine motor scores**						
Sex						
Girls	0.488 (1.384)	.73	0.629 (1.367)	.65	0.841 (1.335)	.53
Boys	0 [Reference]	NA	0 [Reference]	NA	0 [Reference]	NA
Age	0.198 (0.388)	.61	0.282 (0.382)	.46	0.082 (0.378))	.83
Maternal age at birth	0.122 (0.098)	.22	0.107 (0.099	.28	0.073 (0.099	.46
Household income	0.236 (0.853)	.78	0.319 (0.838))	.70	−0.274 (0.841)	.75
Birth weight	NA	NA	4.284 (1.572)	.008	0.956 (1.950)	.63
Gestational age	NA	NA	−0.405 (0.411)	.33	−0.388 (0.401)	.34
Head circumference at birth	NA	NA	NA	NA	1.359 (0.487)	.007
**Gross motor scores**						
Sex						
Girls	−0.590 (1.745)	.74	−0.529 (1.754)	.76	−0.165 (1.747)	.93
Boys	0 [Reference]	NA	0 [Reference]	NA	0 [Reference]	NA
Age	−0.292 (0.489)	.55	−0.218 (0.491)	.66	−0.367 (0.495)	.46
Maternal age at birth	0.000 (0.123)	>.99	−0.049 (0.127)	.70	−0.053 (0.129)	.68
Household income	1.335 (1.076)	.22	1.537 (1.076)	.16	1.094 (1.100)	.32
Birth weight	NA	NA	4.284 (2.017)	.04	1.291 (2.551)	.61
Gestational age	NA	NA	−0.492 (0.528)	.35	−0.432 (0.524)	.41
Head circumference at birth	NA	NA	NA	NA	1.122 (0.638)	.08

Abbreviation: NA, not applicable.

### Cognitive

The mean (SD) age equivalent for the cognitive domain was 2.24 (3.09) months, with a range from 16 days to 19 months. Most children (89 [73.8%]) demonstrated cognitive skills reflecting an ability to take in and respond to their environment (eg, recognizing caregiver, responding to sounds). Nearly one-quarter (25 [20.4%]) were able to engage with and explore objects, but fewer than 10% (12 [9.7%]) demonstrated emerging problem-solving skills (eg, finding a fallen object).

Almost all children (118 [97.5%]) scored at the standard score floor of 55 on the cognitive domain, with mean score of 55.3 and minimal variability (SD, 1.8; range 55-70). Raw scores resulting in the standard score of 55 ranged from 1 to 42.

Head circumference at birth was the only factor associated with cognitive raw scores in the regression analysis. The positive linear association between head circumference and cognitive scores of 0.376 (*P* < .001) was confirmed. Multiple linear regression analysis results indicated that as head circumference at birth increased by 1 cm, cognitive domain scores were 1.4 points higher (*P* < .04), holding all other factors constant. The collective factors (age, sex, maternal age at birth, birth weight, household income, gestational age, and head circumference) explained approximately 16.8% of the variability in cognitive scores (*F*_7,91_ = 3.19; *P* = .005).

### Motor Composite

Children received a mean standard score of 46.26 on the motor composite (SD, 1.83; range, 46-64). Nearly all (116 [95.9%]) scored a 46, the floor of the motor scale. Raw scores between 0 and 54 resulted in a standard score of 46.

#### Fine Motor Scale

The fine motor domain tended to be lower than all other domains, with a mean age-equivalent score of 2.15 months (SD, 2.93 months; range, 16 days to 20 months). More than half of children (68 [56.3%]) were able to follow a ring with their eyes and almost half (55 [45.6%]) with their head, and 62 (51.5%) could hold onto the ring when it was placed in their hands. However, only 32 (26.2%) were able to keep their hands open, 22 (18.5%) were able to rotate their wrists, and 9 (7.8%) could independently grasp items. Head circumference was associated with fine motor scores (β = 1.359; SE, 0.487; *P* < .007) based on the multiple regression analysis, and the model with the collective predictors explained 10.5% of the variance (*F*_7,91_ = 2.64; *P* = .02).

#### Gross Motor

The mean (SD) age equivalent for gross motor was 2.29 (2.52) months, with a range of 16 days to 15 months. Only 2 children (1.7%) walked independently, and 2 (1.7%) were mobile through crawling. Most were not ambulatory and demonstrated little control over either upper or lower limbs. Overall, 9 children (7.8%) could sit unsupported, and 4 (3.5%) could roll from back to stomach or stomach to back. More than half (74 [61.1%]) exhibited head control while upright, in dorsal and ventral suspension, and while being carried. Head circumference at birth was positively correlated with gross motor skills (*r* = 0.236; *P* < .001). The multiple linear regression indicated that head circumference at birth was a significant factor in gross motor scores (β = 1.12; SE, 0.64; *P* = .08); however, the overall model did not fit.

### Language Composite

There was more variability in overall language scores than in cognitive or motor scores. The mean (SD) standard score was 47.7 (2.89), with a range of 47 to 71. Overall, 106 (87.6%) scored at the floor of 47 on the language domain. Scores between 0 and 18 resulted in a standard score at the floor.

#### Receptive Language Scale

The mean (SD) receptive language age-equivalent score was 3.02 (2.89) months, with a range of 16 days to 21 months. Receptive language age-equivalent scores were significantly higher than most other domains (cognition: *t* = 3.73; *P* < .001; fine motor: *t* = 6.99; *P* < .001; gross motor: *t* = 2.70; *P* = .008). Early receptive language skills reflect the ability to respond to sounds in the environment, skills most children were able to demonstrate. Overall, 43 children (35.9%) demonstrated more abstract receptive language, such as responding differentially to their name, while only 1 (0.83%) could identify objects based on words. Birth weight (β = 2.2; SE, 0.92; *P* = .02) and maternal age at birth (β = 0.11; SE, 0.05; *P* = .02) were significant factors associated with receptive language scores in the model with all factors. The collective factors explained approximately 9.5% of the variability in scores (*F*_7,91_ = 2.48; *P* = .02).

#### Expressive Language

Mean (SD) expressive language age-equivalent scores were 2.30 (2.52) months range (range, 16 days to 15 months), and scores were significantly higher than fine motor scores (*t* = 5.23; *P* < .001). Only 2 children (1.9%) were using words to communicate, and 9 (7.8%) were babbling and imitating sound approximations. Overall, 87 (71.8%) could vocalize moods and smile, and nearly three-fourths (85 [70.9%]) could laugh or vocalize in response to social interaction. Overall, 47 (38.8%) were using vowel sounds. There was no significant positive association between the collective factors in the linear regression model and expressive language scores.

## Discussion

To our knowledge, this study is the largest report to date of children with obvious birth defects as a result of ZIKV infection. Children with the most severe cases of CZS were expected to have substantial delays and impairments,^11,23^ but the magnitude of delay and relative strengths and weaknesses has not been previously documented.

We found profound delays in all developmental domains. At age 2.5 years, most children were functioning more like a child aged 2 to 3 months. Most scored at the floor of the standardized scales, although we found a wide range of raw scores associated with the floor.^24,25^ Because we are unable to meaningfully interpret analyses based on standard scores owing to the lack of variability, we used age-equivalent scores and raw scores to examine within- and between-participant variance and describe developmental profiles.

We observed a relative strength in receptive language. Given the significant motor and visual impairments, these children’s primary means for interacting with their environment appears to be through auditory input. Most could take in and respond to the environment, react to sounds and voices, differentiate between familiar and unfamiliar voices, and respond to their names. They also interacted vocally with their environment, making sounds to convey moods and engage in social interactions. This relative strength could provide important direction for intervention and support.

Encouragingly, maternal age was positively associated with receptive language, suggesting a potential environmental influence on outcomes. While we do not have data yet to confirm this hypothesis, it is possible that older mothers talk more often and provide more verbal stimulation to their children, resulting in higher receptive language scores. This is an important area for future research.

In contrast to language outcomes, we found more substantial motor impairments. Previous studies have suggested that many children with CZS meet criteria for cerebral palsy,^26^ and arthrogryposis, or congenital joint contractures, is a well-documented comorbidity in CZS.^27^ Intentional use of hands was a significant challenge for most children.^26,27^ This finding is consistent with earlier studies of children with ZIKV-associated microcephaly. More refined assessments are needed to understand the full extent of motor impairments. For example, previous studies have documented abnormal persistence of primitive reflexes^12^ and motor functioning in the severe level on the Gross Motor Function Classification System.^28^ We will assess these motor variables longitudinally to measure progression or regression over time.

Because most children did not have functional use of hands or the ability to control movement in their limbs, they were unable to demonstrate higher-order cognitive skills, such as object permanence or basic problem-solving. Novel techniques, such as heart-defined attention^29^ or habituation procedures,^30^ may be necessary to assess the extent to which children with severe CZS are capable of learning from their environment.^29^

The extent of microcephaly, as defined by head circumference at birth, was the only significant factor associated with cognitive and motor outcomes. More than 60% of children had head circumferences more than 3 SDs below the mean at birth. Nearly all also had significant brain calcifications. Even with no microcephaly or other birth defects, higher-than-expected rates of brain and ocular anomalies have been reported among those with laboratory evidence of ZIKV infections.^31,32^ These findings suggest that neurodevelopmental differences are likely to manifest as children get older, even in those without obvious challenges in infancy. Indeed, Neilsen-Saines et al^14^ reported that approximately one-third of children showed significant delays on the BSID-III, which included only 6 participants with unresolved microcephaly. Interestingly, in this cohort with less impairment, language function was more delayed than cognitive or motor function, the opposite of our findings. This may reflect a compensation of auditory pathways in children whose vision and motor skills are significantly challenged.

### Limitations and Strengths

This study highlights the weaknesses and strengths of using the BSID-III. Standard scoring procedures are not likely to provide useful information beyond demonstrating the extent of developmental impairment in children with CZS. Furthermore, although items span the full developmental spectrum from birth through toddlerhood, the reliance on visual and motor output may penalize children with CZS in demonstrating what they can do. Conversely, the BSID-III raw scores and age-equivalent scores provide a sensitive measure of potential change over time, allowing for monitoring of gain or loss of skills in response to time, treatment, or seizures. Additionally, adapting the BSID-III to accommodate visual and motor difficulties made it possible to more accurately assess the developmental function of children with CZS. However, there are few appropriate, validated assessment tools that are appropriate for children with such a range of functional skills.

Two other limitations should be noted. First, standards for confirming ZIKV have improved over the last 2 years, and so we do not have criterion-standard methods for ZIKV testing on our participants. Furthermore, although this is a large cohort of children with CZS, they were all ascertained from 1 center in Brazil; therefore, we are limited in our ability to generalize findings to other cohorts of affected children who may have different parent demographic characteristics or access to care.

## Conclusions

The 2015 ZIKV outbreak in northeastern Brazil left nearly 3000 children with significant brain injury and severe comorbid impairments. Although the outbreak is no longer considered an epidemic, 447 confirmed cases of ZIKV in pregnant women were reported in 2019, suggesting that ZIKV continues to pose a threat.^33^ While scientists work on vaccines to prevent future outbreaks and on vector control to reduce the spread of infected mosquitos, we found that affected children, their families, and society face a lifetime of challenges, with associated uncertainty about the future. Understanding comorbid conditions and developmental profiles can help to develop targeted interventions to support children and their families. We will continue to monitor outcomes, explore alternative ways to assess abilities, document burden, and assist in treatment planning, hopefully providing information to help maximize development and quality of life for affected children and their caregivers.
